# Kussmaul's Sign Provoked by Intracardiac Metastasis of Endometrial Neuroendocrine Tumor

**DOI:** 10.1002/kjm2.70026

**Published:** 2025-04-14

**Authors:** Yi‐Hsun Chen, Chin‐Chuan Chang, Yao‐Kuang Wang, I‐Chen Wu

**Affiliations:** ^1^ Division of Gastroenterology, Department of Internal Medicine Kaohsiung Medical University Hospital, Kaohsiung Medical University Kaohsiung Taiwan; ^2^ School of Post Baccalaureate Medicine College of Medicine, Kaohsiung Medical University Kaohsiung Taiwan; ^3^ School of Medicine College of Medicine, Kaohsiung Medical University Kaohsiung Taiwan; ^4^ Department of Nuclear Medicine Kaohsiung Medical University Hospital, Kaohsiung Medical University Kaohsiung Taiwan

We present the case of a 66‐year‐old woman with a history of type 2 diabetes and mitral valve regurgitation who presented with vaginal bleeding and was diagnosed with a grade 3 (Ki‐67: 15%–25%) endometrial neuroendocrine tumor (NET) in 2022. Despite undergoing surgery and chemotherapy, the tumor recurred with multiple metastases and she has received further chemotherapy and targeted therapy. She subsequently developed exertional dyspnea, abdominal distension, and lower limb edema. Notably, her jugular venous pressure paradoxically increased during inspiration, consistent with Kussmaul's sign. Her heart rhythm was irregular; there was a systolic murmur at the apex and mild crackles in bilateral low lung fields. A computed tomography (CT) scan of the abdomen revealed multiple tumors in the lung, heart, peritoneum, and massive ascites. Cardiac echocardiography revealed severe mitral regurgitation and a mass approximately 3 cm in diameter in the right ventricle.

The patient subsequently underwent PET/CT scans with F‐18 FDG and Ga‐68 DOTATOC. On the maximal intensity projection (MPI) of F‐18 FDG PET (Figure 1A), there were hypermetabolic metastatic lesions in bilateral lung fields (SUVmax 5.08), hepatic hilar, peritoneal mass, intraabdominal lymph nodes (SUVmax 11.15), and notably the heart (SUVmax 13.46). On the Ga‐68 DOTATOC PET image (Figure 1B), multiple lesions with higher uptake compared to that in FDG PET were noted in bilateral lungs (SUVmax 12.38) and intraabdominal lymphadenopathies (SUVmax 24.36). Mild to moderate grade of Ga‐68 DOTATOC uptake was noted in the heart with SUVmax 8.36 (Figure 1D); however, the uptake is lower than that in FDG PET with SUVmax 13.46 (Figure 1C). The Krenning score for somatostatin receptor PET imaging was 3.

**FIGURE 1:**
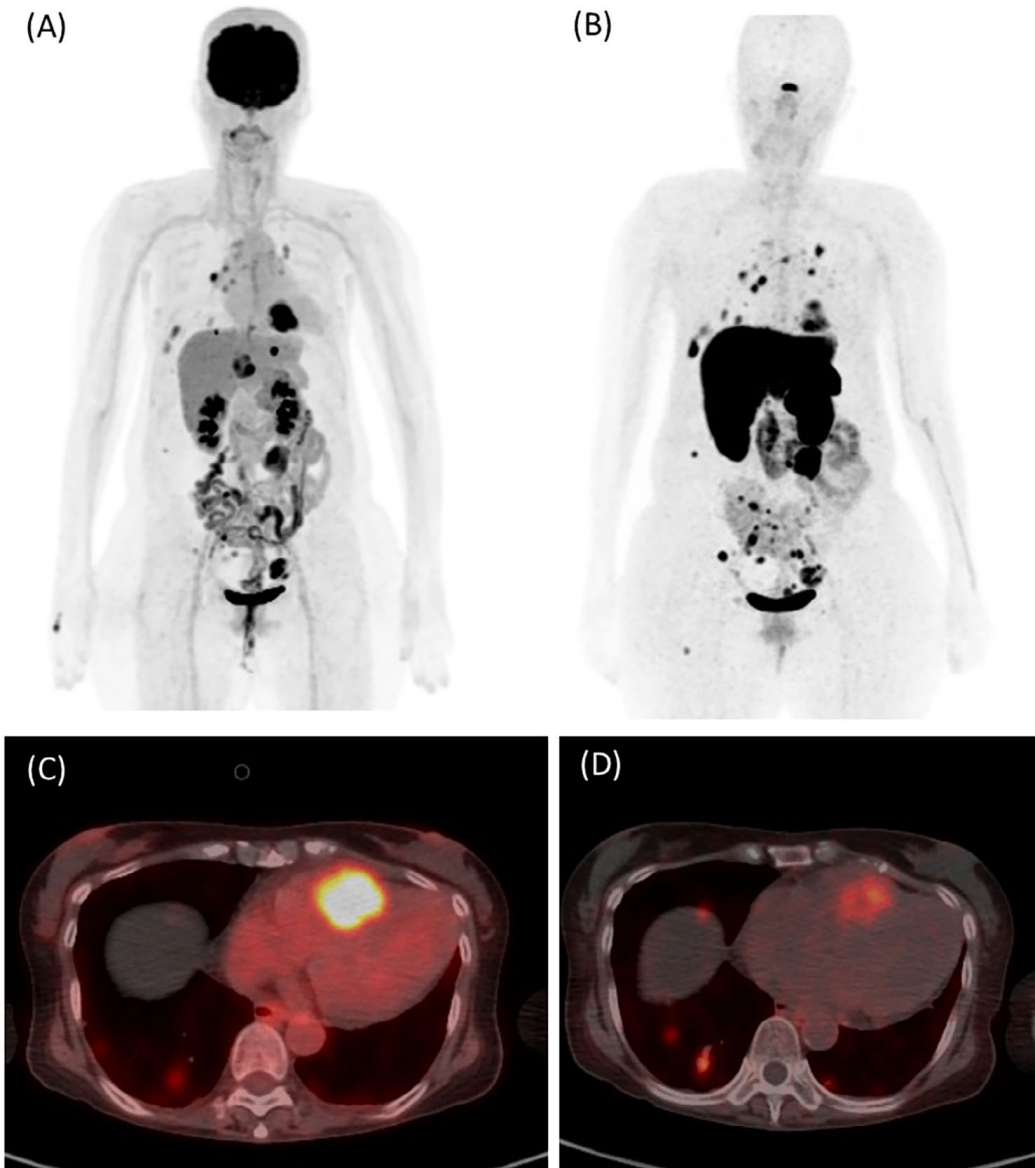
On the maximal intensity projection (MIP) of F‐18 FDG PET (A), hypermetabolic metastatic lesions were observed in both lung fields, the hepatic hilum, a peritoneal mass, intra‐abdominal lymph nodes, and, notably, the heart. In the Ga‐68 DOTATOC PET image (B), multiple lesions with higher uptake compared to FDG PET were identified in both lungs and intra‐abdominal lymphadenopathies. The intracardiac tumor exhibited high FDG uptake (SUVmax 13.46, [C]), while Ga‐68 DOTATOC uptake was mild to moderate (SUVmax 8.36, [D]).

Only a few case reports have described intracardiac metastases from NET [1, 2, 3, 4], with an incidence rate of 2%–4.3% [1, 2]. Most of these cases did not exhibit cardiac symptom [4]. Our patient received 2 cycles of peptide receptor radionuclide therapy (PRRT) with Lutetium‐177. Her abdominal distention improved, reducing the frequency of paracentesis. However, dyspnea and neck pulsation persisted. Follow‐up CT revealed regression of the intra‐abdominal lymph nodes, but the cardiac tumor continued to enlarge. She subsequently received supportive care and passed away approximately 2 months later. Our findings align with previous studies suggesting that the NETPET score may serve as a prognostic factor for PRRT [5]. The score predicts a more favorable PRRT response when the lesion shows higher Ga‐68 DOTATOC uptake compared to F‐18 FDG uptake.

## Conflicts of Interest

The authors declare no conflicts of interest.

## Data Availability

Data sharing not applicable to this article as no datasets were generated or analysed during the current study.
